# Porcine placenta hydrolysates regulate calcium disturbance in MC3T3-E1 osteoblastic cells

**DOI:** 10.1186/s12906-016-1202-1

**Published:** 2016-07-25

**Authors:** Hwa-Young Lee, Hyung-Ryong Kim, Sun-Young Park, Han-Jung Chae, Jong-Hyun Kim

**Affiliations:** 1 Department of Pharmacology and New Drug Development Institute, Medical School, Chonbuk National University, Jeonju, 560-182 Republic of Korea; 2 Department of Dental Pharmacology, College of Dentistry, Wonkwang University, Iksan, 570-749 Republic of Korea; 3 CODEBIO CO., LTD, Busong 1gil 62, Jiksan-eup, Seobuk-gu, Cheonan, Chungnam 331-815 Republic of Korea; 4 Department of Obstetrics and Gynecology, Institute for Medical Sciences, Chonbuk National University Medical School, Jeonju, 560-182 Republic of Korea

**Keywords:** Osteoporosis, Oxidative damage, Calcium, Porcine placenta hydrolysates, ER stress

## Abstract

**Background:**

In bone metabolism, Ca^2+^ disturbance and oxidative damage are the main biochemical factors related to pathology. Osteoblasts are bone-forming cells that also control bone endocrinology. Endocrine hormones and proteins are matured, folded, and secreted in the endoplasmic reticulum (ER). ER stress has emerged as a new pathological mechanism to explain bone disturbance. Here we studied the role of porcine placenta hydrolysates (PPHs) in the regulation of ER stress.

**Methods:**

Cell viability was determined in vitro using trypan blue dye exclusion. ER stress and apoptosis were evaluated using immunoblotting and a caspase kit. The fluorescent Ca^2+^-binding dye Fura-2/AM was used to measure changes in intracellular Ca^2+^ ([Ca^2+^]_i_). ROS levels, NADPH oxidase activity, and superoxide dismutase (SOD) activity were also measured.

**Results:**

PPHs protected MC3T3-E1 osteoblastic cells against thapsigargin (Tg)-induced ER stress. Moreover, PPHs regulated caspase-12 and −3 activities, thereby protecting against cell death, and also regulated Tg-induced Ca^2+^ release. The Ca^2+^ chelator BAPT/AM also regulated caspase-12 and −3 activities and prevented Ca^2^ stress-induced cell death. In the presence of PPHs or BAPTA/AM, Ca^2+^-related ROS were also regulated, as demonstrated by alterations in NADPH oxidase and SOD activity.

**Conclusions:**

PPHs appear to regulate bone metabolism disturbance by controlling Ca^2+^ concentrations, and thus ER stress and ROS, in osteoblasts cultured in vitro.

**Electronic supplementary material:**

The online version of this article (doi:10.1186/s12906-016-1202-1) contains supplementary material, which is available to authorized users.

## Background

Osteoporosis is characterized by decreased bone strength, decreased bone mass, and deterioration of bone tissue. An imbalance between bone resorption and bone formation is the dominant mechanism causing osteoporosis [1, 2]. Since new bone formation primarily depends on osteoblasts, factors that disturb their bone-forming characteristics can lead to bone formation defects or related pathological conditions. Osteoblasts are secretory cells with well-developed endoplasmic reticulum (ER) cristae. The balance of osteoblasts and osteoclastic cells is carefully controlled [1, 3, 4]. During severe pathologic stress, apoptosis occurs in osteoblasts, which disturbs the balance between osteoblasts and osteoclasts and ultimately leads to bone resorption and related disease conditions [5].

The ER plays a major role in controlling protein folding and secretion in cells. Various acute and chronic conditions, including misfolded proteins and Ca^2+^ disturbances, can alter ER function and lead to ER stress [6–8]. Ca^2+^ disturbance and oxidative stress (which is related to Ca^2+^ disturbance) have been suggested to lead to pathological ER stress. Furthermore, ER stress has been reported to contribute to several diseases, including bone diseases [9, 10]. Osteoblast apoptosis associated with ER stress is one of the predominant mechanisms of osteoporosis pathogenesis [11–14]. In stressed osteoblasts, endocrine function, including the production of bone formation hormones (e.g., osteopontin and osteocalcin), is damaged [15, 16]. During severe ER stress, apoptosis is also induced [17–19]. Consequently, ER stress regulators are of great importance in bone-related endocrine cells.

The placenta is an organ found exclusively in women during pregnancy that supplies nutrients and oxygen to the developing fetus. The nutritional substances and vitamins therein can be extracted in the form of porcine placenta hydrolysates (PPHs). PPHs are considered to be a reservoir of cytokines, hormones, bioactive peptides, enzymes, growth factors, and minerals [20]. PPHs also contain valuable bioactive compounds that have various biological functions, including inhibiting aging, inflammation, sunburn, gene mutation, and oxidation [21]. PPHs have been used for wound healing in Korean folk medicine [22, 23] and have been demonstrated to have immunomodulatory effects in various studies [24, 25]. Moreover, PPHs have been used in cosmetic and pharmaceutical products for whitening and oxidative stress-induced diseases, respectively [20]. However, the effect of PPHs on bone-related endocrine cells, including osteoblasts, has received comparatively little attention. To determine the role of PPHs in the endocrine system, it is important to determine the effects of PPHs on osteoblasts, a representative endocrine cell associated with stress conditions.

We based our study on the knowledge that osteoblasts are susceptible to Ca^2+^ disturbance and hypothesized that they amplify their signaling to closely related cells. Thus, in this pharmacological study of PPHs, we studied Ca^2+^ disturbance in the context of related ER stress and cell death. We tested the hypothesis that PPHs regulate ER stress by affecting Ca^2+^ homeostasis, leading to cell protection. Our results indicate that PPHs are a novel group of ER stress regulators, at least in bone-forming osteoblasts, with an additional protective role against Ca^2+^ disturbance.

## Methods

### Materials

PPHs were obtained from Codebio Inc. (Cheonan, Republic of Korea). Hydrogen peroxide and thapsigargin were obtained from Sigma Chemical Company (St. Louis, MO, USA). BAPTA/AM was purchased from Invitrogen (Carlsbad, CA, USA). Caspase-3 and −12 activity kits were obtained from BioVision (Mountain View, CA, USA). All other chemicals and reagents used in this study were of reagent-grade quality and were obtained commercially.

### Cell culture and viability analysis

The MC3T3-E1 osteoblast-like cell line (mouse C57BL/6 calvaria, subclone 4, ATCC No. 58078614) was purchased from the American Type Culture Collection (ATCC; Manassas, VA, USA). MC3T3-E1 osteoblastic cells were cultured in minimum essential medium (α-MEM) supplemented with 10 % fetal bovine serum (FBS), 100 U/mL penicillin, and 100 μg/mL streptomycin (Gibco). Cells were maintained at 37 °C in a humidified atmosphere of 5 % CO_2_. After the cells were cultured with PPHs and/or other agents, cell viability was assessed by trypan blue dye exclusion using a hemocytometer.

### Quantification of apoptosis

To visualize nuclear morphology, cells were fixed in 4 % paraformaldehyde and stained with 2.5 μg/ml Hoechst 33342 DNA dye. Cells with uniformly stained nuclei were scored as healthy and viable. Cells with condensed or fragmented nuclei were scored as apoptotic. To ensure that the counting was unbiased, all petri dishes were coded before the cells were scored. Separately, the apoptosis assay using flow cytometry was performed according to the vendor’s protocol (BD Pharmingen, BD Biosciences, San Jose, USA). Briefly, the cells were treated with 0.1 μM Tg at 37 °C in the presence or absence of 100 μg/mL PPHs or 2 μM BAPTA/AM for 24 h and were trypsinized, washed in PBS and resuspended (1x10^6^ cells/ml) in binding buffer (10 mM HEPES, pH 7.4, 140 mM NaCl, 2.5 mM CaCl_2_). A fraction (100 μl/1x10^5^ cells) of the cell suspension was incubated with 5 μl Annexin V conjugated to FITC and 5 μl propidium iodide (PI) for 15 mins at 25 °C in the dark. 400 μl of binding buffer was added to the suspension and apoptosis was measured immediately using a BD FACSCalibur flow cytometry (BD Biosciences, Franklin Lakes, NJ, USA).

### Immunoblotting

For immunoblotting, MC3T3-E1 osteoblastic cells were lysed by the addition of lysis buffer [50 mM Tris–HCl (pH 7.4), 150 mM NaCl, 0.25 % sodium deoxycholate, 1 % NP-40, 1 mM ethylenediaminetetraacetic acid (EDTA), 0.1 % sodium dodecyl sulfate (SDS), protease inhibitor cocktail set III (EMD Biosciences, La Jolla, CA, USA) and phosphatase inhibitor cocktail set II (EMD Biosciences)] directly onto the cells. The proteins in the lysates (40 μg) were resolved on polyacrylamide gels and transferred to nitrocellulose membranes that were then blocked with skim milk for 1 h at room temperature. The blots were probed overnight at 4 °C with the appropriate primary antibodies, washed, and probed again with species-specific secondary antibodies coupled to horseradish peroxidase (GE Healthcare, Piscataway, NJ, USA). Chemiluminescence reagents (GE Healthcare) were used for signal detection. Primary antibodies included rat anti-GRP78, rabbit anti-GADD153/C/EBP homologous protein (CHOP), rabbit-anti-PERK, mouse anti-eIF2α, rabbit anti-ATF6α, and mouse anti-β-actin (Santa Cruz Biotechnologies, Inc., Santa Cruz, CA, USA), in addition to rabbit anti-phospho-eIF2 and rabbit anti-IRE1α (Cell Signaling Technologies, Inc., Danvers, MA, USA).

### Calcium analysis

The procedures for Ca^2+^ measurements were modified from Kim et al. [26]. Briefly, the low affinity fluorescent Ca^2+^ dye Fura-2/AM (1-[2-(5-carboxyoxazol-2-yl)-6-aminobenzoFURAn-5-oxy]-2-(2-amino-5-methylphenoxy)-ethane-*N*, *N*, *N’*, *N’*-tetraacetic acid pentaacetoxymethyl ester; Molecular Probes, Eugene, OR, USA) was used to measure changes in intracellular Ca^2+^ ([Ca^2+^]_*i*_). Cells were incubated with Fura-2/AM (2 μM) for 30 min at room temperature in Hanks’ balanced salt solution. After loading, cells were washed three times in isotonic buffer without Ca^2+^ (KH buffer: 132 mM NaCl, 5 mM KCl, 10 mM glucose, 10 mM HEPES, and 1.05 mM MgCl_2_). Cells were then promptly treated with thapsigargin. Changes in [Ca^2+^]_*i*_ were determined by measuring the ratio of 340/380 nm excitation (512 nm emission) using an integrated spectrofluorometer (Photon Technology International, Birmingham, NJ, USA). Ca^2+^ concentrations were calculated using the equation [Ca^2+^]_*i*_ *= K*_*d*_(*F*_380__max_/*F*_380__min_)(*R - R*_min_)/(*R*_max_ - *R*); a *K*_*d*_ value of 229 nM was assumed for the binding of calcium to Fura-2/AM. *R*_max_ and *R*_min_ were determined in each experimental group by the consecutive addition of 30 μM Triton X-100 (*R*_max_) and 50 mM EGTA (*R*_min_).

### Measurement of caspase-3 activity

To analyze caspase-3 activity, cell pellets were resuspended in lysis buffer [25 mM HEPES (pH 7.4), 0.1 % Triton X-l00, 10 % glycerol, 5 mM DTT, and a protease inhibitor cocktail] and spun by centrifugation at 13,000 rpm at 4 °C for 30 min. The soluble protein fraction (40 μg) was mixed with 100 μM of the caspase-3-specific substrate Ac-DEVD-AFC (Acetyl-Asp-Glu-Val-Asp-AFC, Sigma-Aldrich) in a final volume of 100 μL and incubated at 37 °C. Caspase-3 activity was analyzed continuously by monitoring fluorogenic AFC release at 37 °C. Subsequently, substrate cleavage was monitored at 405 nm using a SPECTRAmax 340 microplate reader. All data were analyzed using SOFTmax PRO software (Molecular Devices, Sunnyvale, CA, USA).

### Measurement of caspase-12 activity

Caspase-12 activity was measured spectrophotometrically by detecting free AFC cleavage with caspase-12-specific substrates using a Caspase-12 Assay Kit (Biovision, San Francisco, CA, USA). After the lysates were incubated with ATAD-AFC (AFC: 7-amino-4-trifluoromethyl coumarin) for 2 h at 37 °C, the absorbance of each sample at 505 nm was read.

### NADPH oxidase activity assay

Cells were seeded in six-well plates and cultured for 48 h. Next, the cells were treated with 0.1 μM Tg for 24 h in the presence or absence of 100 μg/mL PPHs for 30 min. NADPH oxidase activity was determined based on superoxide-induced lucigenin photoemission, as described by Rao and Maddala et al. [27]. Enzymatic assays were performed in a final volume of 0.2 ml containing 50 mM phosphate buffer (pH 7.0), 1 mM EGTA, 150 mM sucrose, 0.5 mM lucigenin, 0.1 mM NADPH, and cell lysis solution. Enzymatic reactions were initiated by the addition of lucigenin. Photoemission, expressed as relative light units, was measured every minute for 10 min using a luminometer. Assays were performed in the dark at room temperature with all appropriate controls.

### Superoxide dismutase (SOD) activity assay

Cells were seeded in six-well plates and cultured for 48 h. The cells were then treated with 0.1 μM Tg for 24 h in the presence or absence of 100 μg/mL PPHs for 30 min. Next, cells were harvested and the level of SOD activity was determined using a SOD assay kit (k335-100, Biovision) according to the manufacturer's instructions.

### DCFDA assay (ROS production)

The cellular ROS level was measured by following the protocol described by Badham et al. (2010) [28]. Briefly, cells were treated with 0.1 μM Tg at 37 °C in the presence or absence of 100 μg/mL PPHs or 2 μM BAPTA/AM for 24 h. Next, cells were incubated with 10 μM 2’, 7’-dichlorofluorescein diacetate (DCFDA) at 37 °C for an additional 30 min. The fluorescence intensity of 2’,7’-dichlorofluorescein, a product of the reaction between DCFDA and cellular ROS, was analyzed using a fluorescence reader (SpectraMax 190, Molecular Devices, LLC, Sunnyvale, CA, USA).

### Statistical analysis

Results are presented as means ± SEs of *n* cells. Paired and unpaired Student's *t*-tests were applied to the test and control conditions where appropriate. Microcal Origin software (Northampton, MA, USA) was used for all statistical calculations.

## Results

### PPHs alleviate Tg-induced cell death in MC3T3-E1 osteoblastic cells

**Fig. 1:**
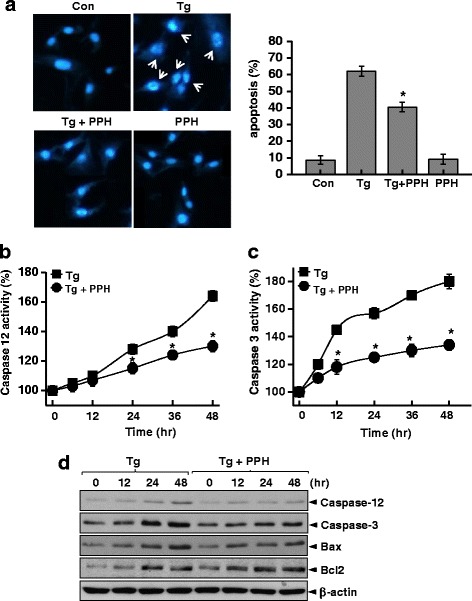
PPHs protect against Tg-induced apoptosis in MC3T3-E1 osteoblastic cells. **a** Hoechst 33342 staining was performed in cells treated with 0.1 μM Tg in the presence or absence of 100 μg/mL PPHs for 24 h. Arrows identify cells with condensed or fragmented nuclei characteristic of apoptosis. Apoptotic cells were quantified based on nuclear condensation or fragmentation (right). Caspase-12 (**b**) and −3 activities (**c**) were analyzed in MC3T3-E1 cells treated with 0.1 μM Tg in the presence or absence of 100 μg/mL PPHs for 0, 12, 24, 36, or 48 h. **d** Cells were treated with 0.1 μM Tg in the presence or absence of 100 μg/mL PPHs for 0, 12, 24, or 48 h. Immunoblotting was performed with antibodies against caspase-12, caspase-3, Bax, Bcl2, and β-actin. ^*^ *p* < 0.05, significantly different from the Tg-treated condition. Tg, thapsigargin; PPHs, porcine placenta hydrolysates

Porcine placenta hydrolysates (PPHs) have traditionally been used to treat bone resorption, especially in menopausal women. Since calcium disturbance is a known mechanism of bone dysmetabolism [29], thapsigargin (a Ca^2+^-ATPase inhibitor and Ca^2+^-disturbing agent) was applied to PPHs-treated or non-treated MC3T3E-1 osteoblastic cells. MC3T3-E1 osteoblasts were used to study the efficacy and function of PPHs on osteoblasts because these secretory cells have highly developed ER. First, we tested the effect of PPHs on cell viability. At concentrations ranging from 25 to 100 μg/mL, PPHs did not significantly affect the viability of MC3T3-E1 osteoblastic cells (Additional file 1: Figure S1A). However, treatment of cells with 0.025, 0.05, or 0.1 μM Tg for 24 h significantly increased cell death in a concentration-dependent manner (Additional file 1: Figure S1B). Interestingly, PPHs significantly blunted Tg-induced cell death in a concentration-dependent manner (Additional file 1: Figure S1C). The kinetics of PPHs-mediated protection (100 μg/mL PPHs) are shown in Additional file 1: Figure S1D. To investigate the mechanism of cell death, apoptosis was analyzed through Hoechst 33342 staining. Representative photomicrographs of MC3T3-E1 nuclear morphology are shown in Fig. 1a. Tg treatment induced nuclear condensation and fragmentation, both of which are characteristic of apoptosis. However, pretreatment with PPHs markedly attenuated this effect. Caspase-12 activation is known to be associated with ER stress-induced apoptosis [30]. Thus, we investigated the effect of PPHs on caspase-12 activation. We found that caspase-12 activity increased significantly after Tg treatment, whereas treatment with PPHs markedly reduced Tg-induced caspase-12 activity in MC3T3-E1 cells (Fig. 1b). Similarly, Tg treatment enhanced caspase-3 activation, and this increase was blocked by PPHs treatment in MC3T3-E1 cells (Fig. 1c). Next, MC3T3-E1 cells were treated with Tg in the presence or absence of PPHs and the levels of various apoptosis-related proteins (caspase-12, −3, Bax, and Bcl-2) were analyzed by immunoblotting. These experiments showed that Tg significantly increased the protein levels of caspase-12, caspase-3, Bax, and Bcl-2 in a time-dependent manner. Interestingly, combined treatment with PPHs and Tg resulted in less increased protein levels of caspase-12, caspase-3, and Bax in MC3T3-E1 cells, rather than further increasing the protein level of Bcl-2, an anti-apoptotic protein. This is an interesting finding because it contrasts with the results obtained with treatment with Tg alone (Fig. 1d). These findings suggest that PPHs protect osteoblasts against Ca^2+^ stress.

### PPHs protect against ER stress-induced apoptosis in MC3T3-E1 osteoblastic cells

**Fig. 2:**
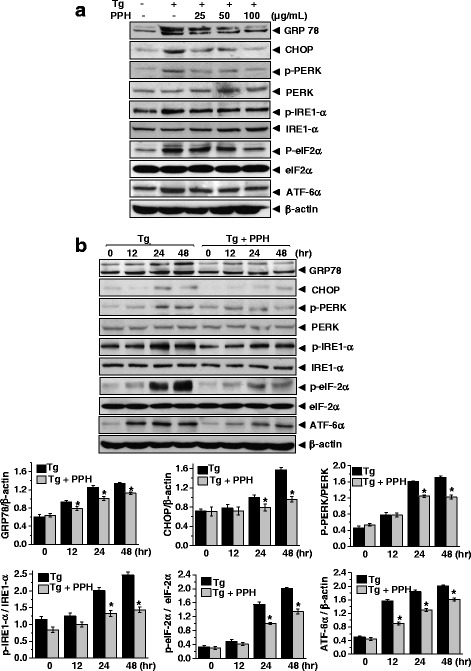
PPHs regulate Tg-induced ER stress response in MC3T3-E1 osteoblastic cells. **a** MC3T3-E1 cells were treated with 0.1 μM Tg in the presence or absence of 25, 50, or 100 μg/mL PPHs for 24 h. Immunoblotting was performed using antibodies against GRP78, CHOP, p-PERK, PERK, p-IRE1α, IRE1α, p-eIF2α, eIF2α, ATF6α, and β-actin. **b** Cells were treated with 0.1 μM Tg in the presence or absence of 100 μg/mL PPHs for 0, 12, 24, or 48 h. Immunoblotting was performed with antibodies against GRP78, CHOP, p-PERK, PERK, p-IRE1α, IRE1α, p-eIF2α, eIF2α, ATF6α, and β-actin. ^*^ *p* < 0.05, significantly different from the Tg-treated condition. Tg, thapsigargin; PPHs, porcine placenta hydrolysates

Ca^2+^ disturbance is linked to intra-ER Ca^2+^ depletion/alteration, which also affects the ER folding machinery that induce ER stress^6–8^. To investigate the effect of PPHs on ER stress in osteoblasts, MC3T3-E1 osteoblasts were incubated with 0.1 μM Tg to induce ER stress. To confirm induction of the ER stress response, we evaluated the expression and phosphorylation status of glucose response protein 78 (GRP78), which is a representative chaperone protein, and C/EBP homologous protein (CHOP), which is a proapoptotic ER stress protein. We also assessed the expression and phosphorylation status of PKR-like ER kinase (p-PERK) and its downstream target eukaryotic initiation factor 2 alpha (eIF2α), which are related to protein translation attenuation, inositol-requiring enzyme 1 (IRE1-α), which has an endonuclease domain and a trans-autophosphorylation kinase domain, and activating transcription factor 6 (ATF6α), which is a transcription factor that activates the transcription of ER molecules. The levels of all these proteins were significantly increased in cells treated with Tg. However, treatment with PPHs inhibited the Tg-mediated increases in the levels of GRP78, CHOP, p-PERK, p-eIF2α, p-IRE1-α, and ATF6-α (Fig. 2a and b), indicating that PPHs affect the regulation of ER stress in the presence of Ca^2+^ disturbances.

### PPHs regulate calcium release and ER stress-mediated apoptosis in MC3T3-E1 osteoblastic cells

**Fig. 3:**
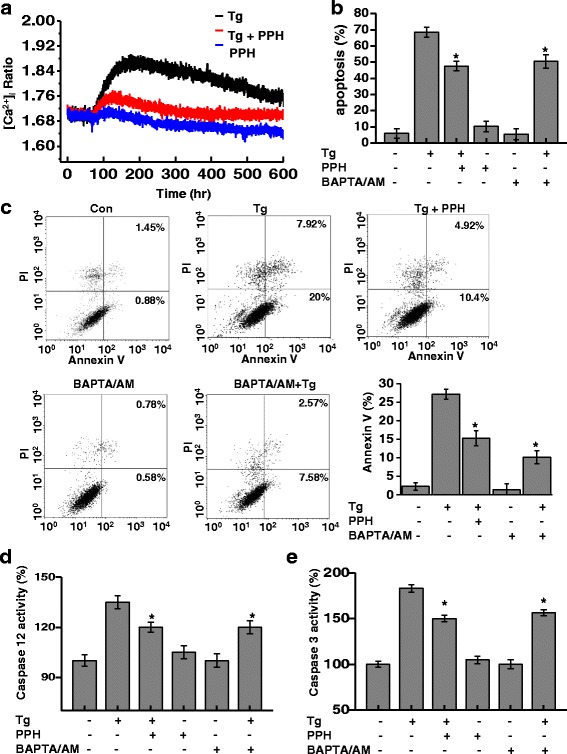
PPHs regulate ER calcium disturbance and apoptosis. **a** Cells were treated with 0.1 μM Tg (black) for 24 h in the presence or absence of 100 μg/mL PPHs (red), or PPHs alone (blue), and then loaded with 2 μM Fura-2/AM for 30 min at 37 °C. The fluorescence intensity of Fura-2/AM was then measured as described in Materials and Methods. **b** to (**d**) Cells were treated with 0.1 μM Tg for 24 h in the presence or absence of 2 μM BAPTA/AM or 100 μg/mL PPHs for 30 min. Apoptosis (**b**) was quantified based on nuclear condensation and fragmentation. Cells were stained with FITC-conjugated Annexin V and PI, followed by flow cytometric analysis (**c**). Caspase-12 (**d**) and −3 (**e**) activities were analyzed as described in Materials and Methods. ^*^ *p* < 0.05, significantly different from the Tg-treated condition. Tg, thapsigargin; PPHs, porcine placenta hydrolysates

Homeostasis of intracellular Ca^2+^ levels ([Ca^2+^]_i_) is important for osteoblast differentiation [29, 31]. To analyze the regulation of [Ca^2+^]_i_ in osteoblasts, we investigated the effects of PPHs on intracellular Ca^2+^ concentration. While the [Ca^2+^]_i_ was significantly increased in Tg-treated cells, treatment with BAPTA/AM significantly attenuated this Ca^2+^ spike in Tg-treated cells (Fig. 3a). To confirm the relationship between PPHs, Ca^2+^, and apoptosis, Tg-treated osteoblasts were pretreated with the Ca^2+^-chelating agent BAPTA/AM. The effect of PPHs alone was also tested. As shown in Fig. 3b, both PPHs and BAPTA/AM protected osteoblasts against Tg-induced apoptosis. Apoptosis levels were also determined by flow cytometry and expressed in units of mean fluorescence intensity. In the MC3T3-E1 cells, the apoptosis level was 27.15 ± 1.4 after Tg treatment for 24 h, whereas the apoptosis level in the PPHs group was 15.32 ± 2.0. The level in the presence of Ca^2+^ chelating agent was similar to that in the presence of PPHs, indicating that PPHs at least partly regulates Ca^2+^-associated ROS production in the Ca^2+^ disturbing stress (Fig. 3c). Consistently, both agents also significantly blocked Tg-induced caspase-12 and −3 activation (Fig. 3d and e), suggesting that PPHs protect osteoblasts from apoptosis by modulating the levels of Ca^2+^.

### PPHs regulate ROS production, NADPH oxidase activity and SOD activity in MC3T3-E1 osteoblastic cells

**Fig. 4:**
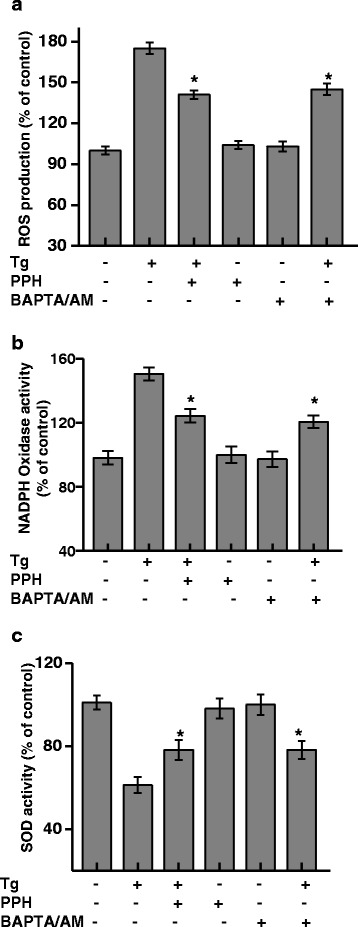
PPHs regulate ROS production, NADPH oxidase activity, and SOD activity in MC3T3-E1 osteoblastic cells. MC3T3-E1 cells were treated with 0.1 μM Tg for 24 h in the presence or absence of 2 μM BAPTA/AM or 100 μg/mL PPHs for 30 min. ROS production was assessed by DCFDA staining (**a**) as described in Materials and Methods. NADPH oxidase (**b**) and SOD activity (**c**) were analyzed. ^*^ *p* < 0.05, significantly different from the Tg-treated condition. Tg, thapsigargin; PPHs, porcine placenta hydrolysates

To analyze the relationship of Ca^2+^ with ROS in the presence of PPHs, we determined the effect of PPHs on Tg-induced ROS release. As expected, treatment with 0.1 μM Tg resulted in significantly increased ROS production. However, exposure to 100 μg/mL PPHs significantly attenuated ROS production in the Tg-treated MC3T3-E1 cells (Fig. 4a).

Next, we examined the effect of PPHs on the activity of NADPH oxidase, an enzyme that produces ROS. As shown in Fig. 4b, PPHs and BAPTA/AM attenuated Tg-induced NADPH oxidase activity. Consistently, Tg significantly suppressed the activity of SOD, a representative antioxidative enzyme that eliminates superoxides. This effect was also attenuated by PPHs and Ca^2+^ chelation (Fig. 4c). These findings indicate that the antioxidant effect of PPHs may contribute to their protective effects in osteoblasts, and are consistent with a model in which PPHs regulate Tg-induced elevations in [Ca^2+^]_i_ and subsequent ROS production in osteoblastic cells.

## Discussion

In this study, we found that PPHs inhibit Ca^2+^ disturbance-related osteoblast death. The basic mechanisms underlying this inhibition include the regulation of Ca^2+^, oxidative stress, and ER stress. We suggest that PPHs contribute to osteoblast-based endocrinal balance, implying that PPHs could potentially be used as therapeutic agents to control bone dysmetabolism.

This study showed that PPHs inhibit the cellular responses triggered by Ca^2+^ disturbance and ER stress, thereby protecting osteoblasts from apoptosis. ER stress has been reported to be involved in apoptosis during various pathophysiological processes, including osteoporosis [11, 12, 29]. ER stress pathways are generally activated in response to various stress conditions, such as the accumulation of misfolded proteins, disturbances of Ca^2+^ homeostasis, and disturbances in energy metabolism [6, 7]. As shown in Figs. 1 and 2, Tg-induced cell death and ER stress were inhibited in PPHs-treated osteoblasts. Our results suggest that PPHs act by inhibiting Ca^2+^ release (Fig. 3a). Both Ca^2+^ disturbance and ER stress have been implicated in the pathogenesis of osteoporosis [11, 12, 29]. Ca^2+^ is an essential intracellular signaling molecule involved in the regulation of numerous cellular processes, including cell proliferation, differentiation, morphology, and function [32]. The intracellular Ca^2+^ concentration can be significantly increased via Ca^2+^ influx from the extracellular space or by Ca^2+^ release from the ER [33]. The release of Ca^2+^ from the ER is mainly regulated by the inositol trisphosphate 3 (IP3) receptors (IP3Rs) and the ryanodine receptors (RyRs) [7]. Moreover, Ca^2+^-activated signaling pathways have been demonstrated to regulate osteoblast proliferation and differentiation [27]. In addition, Ca^2+^ is also involved in the synthesis, folding, and post-translational modifications of proteins in the ER. Disturbance of the Ca^2+^ balance activates the unfolded protein response (UPR) in an attempt to restore homeostasis [34]. The UPR signaling axis, which includes GRP78, CHOP, p-IRE-1α, p-PERK, p-eIF2α, and ATF-6α was highly activated under Ca^2+^ stress, whereas PPHs attenuated the UPR (Fig. 2a, b). Usually, unfolded protein stress in the ER (ER stress) activates the ATF6, IRE-1α, and PERK branches of the UPR. This activation, in turn, regulates the expression of target genes involved in the modulation of ER protein folding, such as GRP78 and XBP1 [35]. Mild ER stress has been demonstrated to aid osteoblast differentiation [36]. However, if the stress is prolonged and unmitigated, the UPR switches to initiate cell apoptosis [19, 37]. Persistent stress in osteoblasts that leads to apoptosis and affects communication with other bone cells is also considered to be ER stress. Ca^2+^ stress seems to be more related with persistent/prolonged stress conditions, whereas PPHs regulate the ER stress response. The in vitro analyses presented here indicate that PPHs contribute to Ca^2+^ maintenance in osteoblasts, leading to ER stress regulation and cell protection. Additionally, we showed that the ER redox balance explains the association with Ca^2+^ disturbance. In studies of Ca^2+^ homeostasis imbalance, ER stress has been highly linked with ER stress-associated ROS [38]. In this study, we hypothesized that ROS might be generated from Ca^2+^ disturbances resulting from Tg-induced ER stress. As expected, treatment with Tg increased ROS levels (Fig. 4a). However, the intracellular ROS content was significantly decreased in PPHs-treated osteoblasts compared with Tg-treated osteoblasts. The relationship between ER-induced oxidative stress and Ca^2+^ disturbance has been investigated [38]. Ca^2+^ can be a combined physiological and pathological effector. Moreover, ROS are generated by various environmental agents as well as during normal cellular metabolism. ROS play a major role in the pathogenesis of various diseases, including osteoporosis [39]. Osteoporosis is characterized by reduced bone mass resulting from an imbalance between bone formation by osteoblasts and bone resorption by osteoclasts. Since the rate of osteoblast apoptosis regulates bone formation [1, 2, 40, 41], the effect of PPHs on ROS may yield a protective effect that inhibits osteoporosis.

In this study, we found that PPHs regulate bone metabolism disturbances in osteoblasts by controlling Ca^2+^ concentrations, thereby also affecting related ER stress and ROS. Since PPHs do not include ovarian hormones, this regulatory effect on bone metabolism disturbance is not associated with ovarian hormones such as estrogen. Thus, improvements in the amino acid profiles of PPHs should be considered, as should the presence of modified amino acids. Nutrients and trace minerals, including essential amino acids such as arginine, lysine, vitamin K, Mn, B, vitamin D, Zn, Cu, folate, and Si are often used to improve bone structure [42]. Dietary arginine and lysine are also believed to play important roles in bone development, growth, and modeling [42, 43]. Arginine is involved in both the synthesis of substrates (polyamine and L-Pro) implicated in collagen synthesis and in the production of growth hormones, including insulin-like growth factor-I [44]. In addition, arginine is thought to alleviate metabolic disturbances in Ca^2+^ absorption, growth, and ossification defects [45]. PPHs contain large amounts of arginine and essential amino acids. Thus, since PPHs contain arginine and other essential amino acids, they may be useful preventive or therapeutic agents against osteoporosis.

## Conclusions

This study suggests that PPHs protect bone-forming MC3T3-E1 osteoblasts against Ca^2+^ stress. In addition, PPHs regulate Ca^2+^ release and the related ROS and ER stress responses. Our data also indicate that PPHs are a new ER stress regulator, at least in bone-forming osteoblasts. The insights from this in vitro study have implications on our understanding of the mechanism by which PPHs might exert therapeutic effects against bone disturbance.

## Abbreviations

ER, endoplasmic reticulum, PPHs, porcine placenta hydrolysates, ROS, reactive oxygen species, Tg, thapsigargin

## Additional file

Additional file 1:
**Figure S1**. Protective effects of PPHs on Tg-induced cell death in MC3T3-E1 osteoblastic cells (PDF 119 KB)
