# Supplementary material for: Porcine placenta hydrolysates regulate calcium disturbance in MC3T3-E1 osteoblastic cells
Source: BMC Complement Altern Med. 2016 Jul 25;16:237. doi: 10.1186/s12906-016-1202-1 (PMC4960880; doi:10.1186/s12906-016-1202-1)

Porcine placenta hydrolysates regulate calcium disturbance in MC3T3-E1 osteoblastic cells

Hwa-Young Lee[a], Hyung-Ryong Kim[b], Sun-Young Park[c], Han-Jung Chae[a], Jong-Hyun Kim[d*]

[a]Department of Pharmacology and New Drug Development Institute, Medical School, Chonbuk National University, Jeonju, 560-182, Republic of Korea, [b]Department of Dental Pharmacology, College of Dentistry, Wonkwang University, Iksan, 570-749, Republic of Korea, [c]CODEBIO CO., LTD, Busong 1gil 62, Jiksan-eup, Seobuk-gu, Cheonan, Chungnam, 331-815, Republic of Korea, [d]Department of Obstetrics and Gynecology, Institute for Medical Sciences, Chonbuk National University Medical School, Jeonju, 560-182, Republic of Korea

*Correspondence to: Jong-Hyun Kim, Department of Obstetrics and Gynecology, Institute for Medical Sciences, Chonbuk National University Medical School, Jeonju, Republic of Korea, Tel: 82-63-250-2395, Fax: 82-63-275-2855, E-mail: obgyn2001@naver.com

Supplementary Figure 1. Protective effects of PPHs on Tg-induced cell death in MC3T3-E1 osteoblastic cells. (A) MC3T3-E1 cells were exposed to PPHs (0, 25, 50, or 100 µg/mL) alone for 24 h and cell viability was assessed by trypan blue dye exclusion. (B) Cells were exposed to Tg (0, 0.025, 0.05, or 0.1 µM) alone for 24 h and cell viability was assessed by trypan blue dye exclusion**.** (C) PPHs was applied to the MC3T3-E1 cells at 0, 25, 50, or 100 µg/mL in the absence or presence of 0.1 µM Tg for 24 h, and cell survival was assessed by trypan blue dye exclusion. (D) Cells were exposed to Tg in the absence or presence of PPHs for 0, 12, 24, 36, or 48 h, and cell viability was assessed by trypan blue dye exclusion**.** $^{*}p < 0.05$, significantly different from the Tg-treated condition. Tg, thapsigargin; PPHs, porcine placenta hydrolysates

Supplementary Figure 1

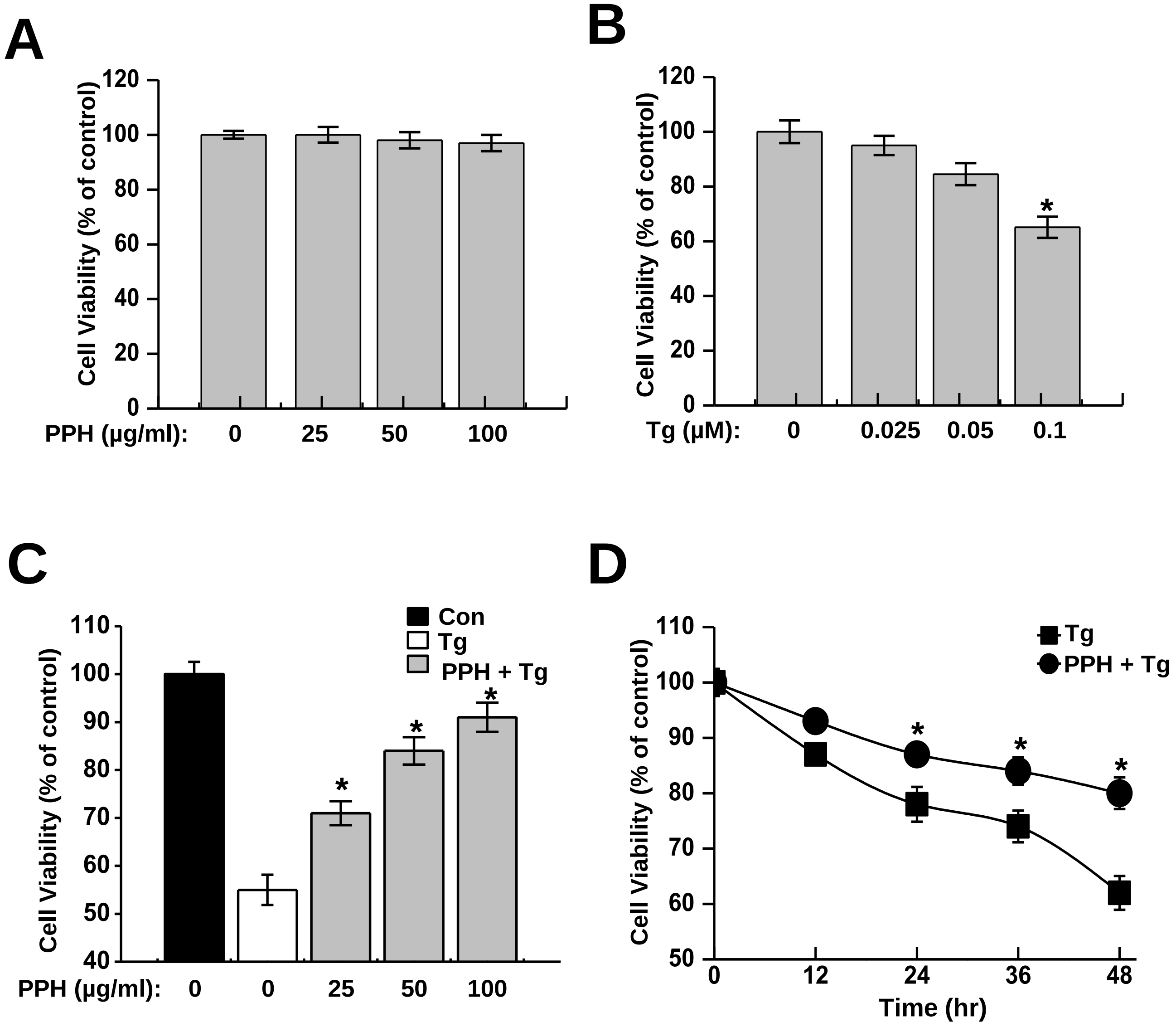

Supplement: Additional file 1: — Figure S1. Protective effects of PPHs on Tg-induced cell death in MC3T3-E1 osteoblastic cells (PDF 119 KB) [file 12906_2016_1202_MOESM1_ESM.pdf]
